# Preparation of Anhydrous Single Crystals of Rare-Earth Halides

**DOI:** 10.6028/jres.067A.036

**Published:** 1963-08-01

**Authors:** Norman H. Kiess

## Abstract

Anhydrous rare-earth halides are prepared by the conversion of the rare-earth oxide to the halide by means of its reaction with the appropriate ammonium halide. Without transfer from the reaction vessel, the halide is melted, then crystallized by slow cooling. The resulting solid usually contains single crystals large enough to permit spectroscopic studies of the compounds.

## 1. Introduction

In recent years much attention has been paid to the optical and magnetic properties of rare-earth compounds. The anhydrous halides have simpler crystal structures [1] 1 than most of the other well- known rare-earth compounds, and yield experimental data that are easier to interpret. It is desirable to have an efficient method for the preparation of single crystals of the anhydrous halides, particularly if one wishes to study intensities of spectral lines as a function of concentration of absorbing ion.

Single crystals of anhydrous rare-earth halides are not difficult to grow from a pure melt. The production of the pure salts has, however, been timeconsuming and difficult. Previous workers [2–8] have prepared them by dissolving the rare-earth oxide in the appropriate acid and subsequent evaporation to crystallize the hydrated halide, followed by dehydration of the hydrated halide in an atmosphere of dry hydrogen halide gas. It has sometimes been necessary, in order to crystallize salts prepared in this way, to purify them by distillation [5, 6]. A much simpler method for the preparation of anhydrous rare-earth halides, except fluorides, was first described by Reed [9] in 1934. None of the early workers [9–12] using this method reported attempts to crystallize their products. In this work it was found that, by using a modification of the basic procedure described by Reed, salts pure enough to crystallize could be produced.

## 2. Method and Apparatus

Anhydrous rare-earth halides can be prepared by the reaction, at high temperatures, of the rare-earth oxide with the appropriate ammonium halide, as represented by the following typical equation:

$${\text{La}}_{2}O_{3}+6{\text{NH}}_{4}\text{Cl}\to 2{\text{LaCl}}_{3}+6{\text{NH}}_{3}+3H_{2}O.$$

**Figure 1:**
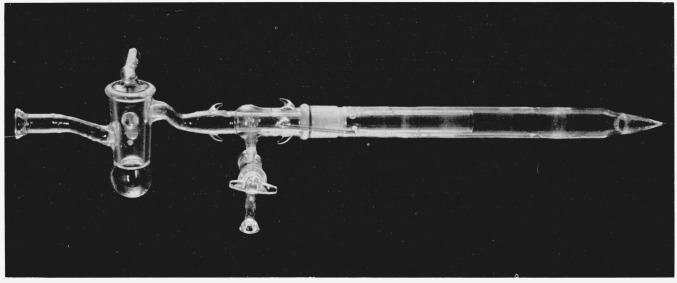
Glassware for preparing crystals. Diameter of vycor tube: 30 mm.

A large excess of ammonium halide must be used. A fourfold stoichiometric excess is adequate for the chloride and the bromide, whereas a tenfold excess is necessary for the iodide. The reagents are ground together with a mortar and pestle until a powdery mixture is obtained, then poured into a vycor tube about 15 in. long and an inch or more in diameter. One end of this tube is drawn to a point and the other is the outer member of a standard taper joint. A stream of dry, inert gas (helium or nitrogen) is to be directed over the mixture. This is effected by the arrangement depicted in figure 1. A Pyrex inner tube extends several inches into the vycor tube. There is a distance of at least an inch between the reacting mixture and the end of the inner tube. A ring seal connects the Pyrex tube with the inner member of the tapered joint. The members of the joint are sealed by means of a silicone O-ring that fits into a groove in the inner member. A small flow of dry gas is started. The assembled reaction vessel is then inserted a sufficient distance into the tube of a horizontal furnace to allow the dry gas to enter the hot zone before passing out through the Pyrex tube. The temperature is raised to the point where the reaction becomes vigorous, which is between 200 and 400 °C, depending on which halide is being made, and is held there until the reaction is nearly complete. The flow of gas carries out the volatile products of the reaction, and the water vapor condenses on the cooler walls of tubing outside the furnace. The appearance and subsequent evaporation of this water is the best guide as to the course of the reaction. The water evaporates about an hour after it appears, indicating that the reaction is close to completion. The temperature is then raised about 100 °C, and is held at this point for several hours, in order to effect completion of the reaction. During this period most of the ammonium halide sublimes and is carried out of the furnace by the flow of gas. Condensation of ammonium halide on the cooler walls of tubing eventually obstructs the flow of gas, but the heating can be interrupted in order to clean and replace the tubing. The residual rare-earth halide is a powder and can react with traces of oxygen and water vapor. The ammonium halide affords protection by reacting with possible decomposition products to regenerate anhydrous rare-earth halide; consequently the last traces of it are removed by evacuating the system. This must be done by careful, simultaneous adjustment of temperature and pumping speed, in order to prevent the rare-earth halide from being blown out of the furnace. When no more ammonium halide condenses on the cooler walls of tubing, the stop-cocks are closed and the evacuated system is transferred to a vertical tube furnace.

The vertical furnace has a heating chamber 6 in. deep and 1½ in. in diameter. A chromel-alumel thermocouple is used to measure temperature in the furnace. There is a sharp temperature gradient at the bottom when power is applied. A saturable reactor in series with the resistance-winding is used to modulate the power. A model JSBG-2 programmer-controller, manufactured by the West Instrument Company, accepts the thermocouple signal and furnishes current for the control-winding of the reactor. By means of this device, control of the time-temperature cycle is achieved. The junction of the thermocouple is placed next to the heating element, about 3 in. from the bottom. The temperature at this point is adjusted to be about 100 °C above the melting-point of the salt. The vycor tube is placed in the furnace so that the tip of the point is below the thermocouple and at a temperature just above the melting-point of the salt. The temperature is then slowly lowered to room temperature over a period of about 18 hr, and the melt solidifies upwards along the axis of the tube.

## 3. Results and Discussion

**Figure 2:**
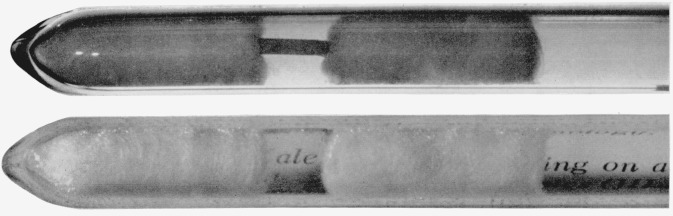
Crystal, mounted in quartz tube, diameter 8 mm, with quartz wool packing. The crystal was cut and mounted by E. F. Williams of Johns Hopkins University.

The procedure described above produced solid masses usually containing regions of single crystal. They could be broken loose from the wall of the tube by tapping it lightly. Slabs of single crystal a centimeter square and a few millimeters thick could be cut, one of which is shown in figure 2. Most of the crystals prepared in this way were halides of lanthanum containing a small amount of optically active rare earth ion as an impurity. The case of samarium impurity in lanthanum halide is particularly interesting because of the two possible oxidation states of samarium in combination with halogens. The absorption spectra of samarium ion in lanthanum bromide prepared by this method proved to be those of triply ionized samarium.^2^

Although it sometimes happened, particularly when preparing iodides, that the resulting solid mass contained no single crystal, it was never found necessary to purify the salt by distilling the melt in order to obtain crystals large enough for spectroscopic work. Single crystals could usually be obtained from polycrystalline masses by grinding them up with a new batch of ammonium halide and repeating the above procedure. It is believed that the factor which is most important in limiting the size of the single crystals is the purity of the melt. The impurities may be due to incomplete reaction with the ammonium halide, reaction with the vycor, or foreign matter in the rare earth oxide. The use of quartz instead of vycor did not produce larger crystals, nor did lengthening the cooling period. As the crystals were large enough for spectroscopic work, these points were not investigated further.

The procedure given here for the synthesis of the anhydrous halides differs from that used by the earlier workers [9–12] in two respects. First, the excess of ammonium halide is greater than that used earlier. Second, the mixture is heated at atmospheric pressure for several hours longer. At the end of this time, most of the ammonium halide has evaporated. The earlier workers removed the ammonium halide by the application of a vacuum after heating at atmospheric pressure for from 1 to 4 hr. The early procedure suffices to produce salts pure enough to give clear solutions in water, hut prolonged heating at atmospheric pressure is necessary to produce salts pure enough to crystallize readily. The immediate melting and subsequent crystallization of the salt follow the removal of the ammonium halide. In view of the extreme instability of these salts it is advisable not to retain them as powders.

The salts melted at temperatures near those commonly given as their melting points. No attempt was made to measure their melting points precisely. The colors exhibited by lanthanum iodide at various temperatures are interesting. The powder at low temperatures is almost white, but as the temperature is raised it turns yellow. The melt is brown and translucent. The crystal at room temperature is light yellowish-green. On the other hand, lanthanum chloride and bromide are almost completely colorless at all temperatures.

An important contribution to this work was the glassblowing and vycor-working of the Glassblowing Shop of the National Bureau of Standards, most of which was done by John Hydro, Jr.

## Footnotes

1 Figures in brackets indicate the literature references at the end of this paper.

2 These spectra were obtained at the Johns Hopkins University under the direction of Dr. G. H. Dieke.
